# Cells derived from iPSC can be immunogenic — Yes or No?

**DOI:** 10.1007/s13238-013-0003-2

**Published:** 2014-01-30

**Authors:** Jiani Cao, Xiaoyan Li, Xiao Lu, Chao Zhang, Honghao Yu, Tongbiao Zhao

**Affiliations:** State Key Laboratory of Reproductive Biology, Institute of Zoology, Chinese Academy of Sciences, Beijing, 100101 China

## Abstract

The induced pluripotent stem cells (iPSCs), derived by ectopic expression of reprogramming factors in somatic cells, can potentially provide unlimited autologous cells for regenerative medicine. In theory, the autologous cells derived from patient iPSCs should be immune tolerant by the host without any immune rejections. However, our recent studies have found that even syngeneic iPSC-derived cells can be immunogenic in syngeneic hosts by using a teratoma transplantation model (Nature 474:212–215, 2011). Recently two research groups differentiated the iPSCs into different germ layers or cells, transplanted those cells to the syngeneic hosts, and evaluated the immunogenicity of those cells. Both of the two studies support our conclusions that some certain but not all tissues derived from iPSCs can be immunogenic, although they claimed either “negligible” or “lack of” immunogenicity in iPSC derivatives (Nature 494:100–104, 2013; Cell Stem Cell 12:407–412, 2013). To test the immunogenicity of clinically valuable cells differentiated from human iPSCs are emergently required for translation of iPSC technology to clinics.

The embryonic stem cells (ESCs), characterized by the capability to both self-renew and differentiate into each cell type, hold great promise for regenerative medicine. However, the ethical concern and immune rejection are the two major hurdles for clinical development of ESCs. The induced pluripotent stem cell (iPSC), developed by Yamanaka group in 2006 through ectopic expression of four reprogramming factors in terminally differentiated somatic cells (Takahashi and Yamanaka, 2006), is believed to be able to get cross those barriers. This breakthrough discovery has greatly reshaped the scientific and political landscapes of stem cell biology. It provides an unprecedented opportunity to model human disease, re-understand the basic biology such as development and differentiation, identify new therapeutic targets and test new therapies etc. Most importantly, the iPSC can potentially provide autologous cell resources in regenerative medicine without concerning the immune rejections led by major histocompatibility complex restriction. It raised the hope that patient-specific iPSCs could become a renewable source of autologous cells for human therapy. Whereas it has been widely predicted that the autologous cells derived from patient-specific iPSCs are immune tolerant in that patient, the immunogenicity of cells derived from iPSC has not been widely examined since the discovery of the iPSCs.

By using a teratoma formation model, we unexpectedly found that some but not all cells derived from mouse iPSC can be immunogenic (Zhao et al., 2011), and the immune rejection response was T cell dependent, supported by the immune rejection was totally blocked in Rag knock-out recipients. Furthermore, in our system we identified two genes (*Hormad1* and *Zg16*), which were abnormally expressed in iPSC-teratoma but not in ES-teratoma, directly contribute to the immunogenicity of iPSC derivatives, supporting by the existence of primed T cells in the mice harboring the iPSC derived teratomas.

The question whether iPSC derivatives are immnogeneic or not is straight forward; however, the answer to this question is very complicated due to the developmental randomness of iPSC and the nondeterminacy of the abnormal expression of the minor antigens. The first hurdle to resolve this question is the hardness to differentiate iPSC into each tissue of our body and test their immunogenicity one by one. The iPSC can produce any kind of tissues in a whole body theoretically, however the directed differentiation of iPSC is still hindered by the low efficiency and it is still impossible to differentiate iPSC into each tissue we need at current stage. The original idea to use teratoma as a model system to test iPSC immunogenicity is that teratoma contains many kinds of tissues differentiated from pluripotent stem cells and it has been successfully used to test the immunogenicity of ES derivatives (Koch et al., 2008). A teratoma is an encapsulated benign tumor harboring many kinds of tissue or organ components resembling normal derivatives of all three germ layers (Tapper and Lack, 1983; Chi et al., 1984), which provide a possibility to probe the immunogenicity of iPSC derived tissues as much as possible in a time. Recently, Dr. Abe’s group studied the immunogenicity of different tissues derived from iPSC including skin cells, bone marrow cells and cardiomyocytes and found that iPSC derived cardiomyocytes *in vitro* but not skin and bone marrow cells are highly immunogenic (For further details, please refer to Sup Fig. 13 in Araki et al., 2013). It is possible that the cardiomyocytes, but not skin and bone marrow cells, harbor the abnormal expressed minor antigens which contribute to the immunogenicity. Another concern is that using the bone marrow transplantation to study the intrinsic immunogenicity of iPCS derivatives is inappropriate, because the hematopoietic stem cells inside the bone marrow can itself develop into different linear of immune cells including regulatory T cells leading to immune tolerance of the graft. In clinic, the pre-transplantation of donor hematopoietic stem cell into the recipients before transplanting the designed organs is routinely used to induce tolerance of allografting. Meanwhile, by differentiation of iPSC into endothelial cells, hepatocytes and neuronal cells, Boyd group studied the immunogenicity of *in vitro* differentiated iPSC derivatives (Guha et al., 2013). Although compared to the counterparts of allografts, the immune rejection response led by syngeneic iPSC descendents was mild, however, the endothelial cells derived from iPSCs showed higher apoptosis rate than syngeneic ES derivatives when cocultured with T cell isolated from endothelial cell experienced syngeneic mice, indicating the iPSC derived endothelial cells can induce immune rejection response (For further details, please refer to Fig. 2E in Guha et al., 2013).

The immunogenicity of iPSC derivatives is very complicated (Fig. 1). It is widely accepted that reprogramming itself can induce both genetic and epigenetic defects in iPSCs (Doi et al., 2009; Kim et al., 2010; Polo et al., 2010; Zhao and Xu, 2010; Gore et al., 2011; Lister et al., 2011). It is possible that those defects can directly or indirectly contribute to the immunogenicity of iPSC derivatives. In supporting of this idea, we have identified a couple of genes overexpressed in iPSC-teratomas, with two genes were confirmed as direct antigens contribute to the immunogenicity of iPSC derivatives. This indicates that not all genes with abnormal expression during differentiation will contribute to the immune rejection responses after transplantation. On the other hand, it is very hard to link the abnormal antigen expression identified in teratomas to an exact cell type, due to the heterogenic structure of teratomas. And the complexity of developmental process makes it difficult to predict which tissue will inherit and present the defects induced by reprogramming during iPSC development and differentiation. So it is not surprising that both Abe and Boyd groups did not detect abnormal gene expression in a specific cell types. A possible solution to this question is to profile the immunogenicity of different tissues as much as possible. For those immunogenic iPSC derived specific tissues, gene expression profiling can be employed to probe the minor antigens contributed to the immunogenicity.

**Figure 1:**
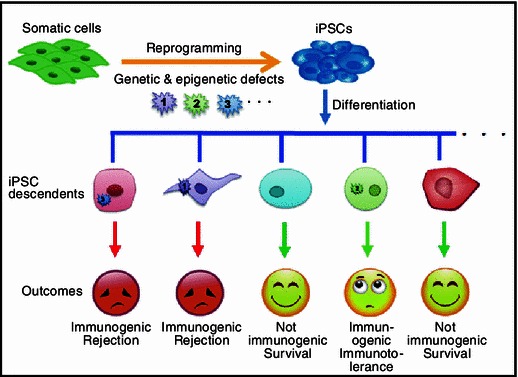
Differential immunogenicity of iPSC derivatives. The genetic and epigenetic defects induced by reprogramming are differentially presented during the iPSC differentiation leading to different consequences. The iPSC descendents without presentation of the defects during differentiation are not immunogenic. Tissues with pronounced defects are immunogenic, but may have distinct destiny

In general, compared to the allograft, the immunogenicity of autologous iPSC derivatives is much weaker, supporting not only by the high teratoma formation rate by B6 iPSCs than allogeneic 129 ES cells in the B6 mice, but also the fact that T cells are only locally but not ubiquitously infiltrated into the teratomas formed by iPSC in B6 mice.

It is noteworthy however, if one certain cell linear differentiated from iPSC expresses immunogenic minor antigens ubiquitously, it still can elicit serious rejection responses, leading to the complete rejection of that tissue (Fig. 1). Which kind of tissues differentiated from iPSCs can be immunogenic and whether they are destined is still an important opening question. Furthermore, it is critical to evaluate the immunogenicity of clinical valuable cells derived from human iPSCs, which can significantly promote the translation of iPSC technology from bench to bedsides.
